# Autoimmune bullous dermatoses in cancer patients treated by immunotherapy: a literature review and Italian multicentric experience

**DOI:** 10.3389/fmed.2023.1208418

**Published:** 2023-07-20

**Authors:** Martina Merli, Martina Accorinti, Maurizio Romagnuolo, Angelo Marzano, Giovanni Di Zenzo, Francesco Moro, Emiliano Antiga, Roberto Maglie, Emanuele Cozzani, Aurora Parodi, Giulia Gasparini, Pietro Sollena, Clara De Simone, Marzia Caproni, Luigi Pisano, Davide Fattore, Riccardo Balestri, Paolo Sena, Pamela Vezzoli, Miriam Teoli, Marco Ardigò, Camilla Vassallo, Andrea Michelerio, Rosanna Rita Satta, Emi Dika, Barbara Melotti, Simone Ribero, Pietro Quaglino

**Affiliations:** ^1^Dermatology Clinic, Department of Medical Sciences, University of Turin, Turin, Italy; ^2^Dermatology Unit, Department of Internal Medicine, Fondazione IRCCS Ca’ Granda Ospedale Maggiore Policlinico, Milan, Italy; ^3^Department of Pathophysiology and Transplantation, Università Degli Studi di Milano, Milan, Italy; ^4^Laboratory of Molecular and Cell Biology, Istituto Dermopatico dell’Immacolata (IDI)-IRCCS, Rome, Italy; ^5^Section of Dermatology, Department of Health Sciences, University of Florence, Florence, Italy; ^6^Section of Dermatology, Department of Health Sciences (DISSAL), University of Genoa, Genoa, Italy; ^7^Dermatology Unit, IRCCS Ospedale Policlinico San Martino, Genoa, Italy; ^8^Dermatology Unit, Department of Surgical and Medical Sciences, Fondazione Policlinico Universitario Agostino Gemelli IRCCS, Rome, Italy; ^9^Dermatology Unit, University Department of Medicine and Translational Surgery, Università Cattolica Del Sacro Cuore, Rome, Italy; ^10^Immunopathology and Rare Skin Diseases Unit, Section of Dermatology, Department of Health Sciences, Azienda Unità Sanitaria Locale Toscana Centro, University of Florence, Florence, Italy; ^11^Section of Dermatology, Department of Health Sciences, Azienda Unità Sanitaria Locale Toscana Centro, University of Florence, Florence, Italy; ^12^Section of Dermatology, Department of Clinical Medicine and Surgery, Università Degli Studi di Napoli Federico II, Naples, Italy; ^13^Division of Dermatology, Outpatient Consultation for Rare Diseases, APSS, Trento, Italy; ^14^Dermatology Unit ASST-Papa Giovanni XXIII, Bergamo, Italy; ^15^Porphyria and Rare Diseases, San Gallicano Dermatological Institute IRCCS, Rome, Italy; ^16^Dermatology Clinic, Fondazione IRCCS Policlinico San Matteo, Pavia, Italy; ^17^Dermatology Unit, Ospedale Cardinal Massaia, Asti, Italy; ^18^Department of Clinical-Surgical, Diagnostic and Pediatric Sciences, University of Pavia, Pavia, Italy; ^19^Department of Medical, Surgical, and Experimental Sciences, University of Sassari, Sassari, Italy; ^20^Melanoma Center, Dermatology, IRCCS Azienda Ospedaliero-Universitaria di Bologna, Bologna, Italy; ^21^Unit of Dermatology, Department of Medical and Surgical Sciences, DIMEC, Alma Mater Studiorum, University of Bologna, Bologna, Italy; ^22^Oncology Unit, IRCCS Azienda Ospedaliero-Universitaria di Bologna, Bologna, Italy

**Keywords:** immunotherapy, anti PD-1, anti PD-L1, cutaneous irAE, bullous pemphigoid, lichen planus pemphigoides, pemphigus, mucous membrane pemphigoid

## Abstract

Cutaneous immune-related adverse events are frequently associated with immune checkpoint inhibitors (ICIs) administration in cancer patients. In fact, these monoclonal antibodies bind the cytotoxic T-lymphocyte antigen-4 and programmed cell death-1/ligand 1 leading to a non-specific activation of the immune system against both tumoral cells and self-antigens. The skin is the most frequently affected organ system appearing involved especially by inflammatory manifestations such as maculopapular, lichenoid, psoriatic, and eczematous eruptions. Although less common, ICI-induced autoimmune blistering diseases have also been reported, with an estimated overall incidence of less than 5%. Bullous pemphigoid-like eruption is the predominant phenotype, while lichen planus pemphigoides, pemphigus vulgaris, and mucous membrane pemphigoid have been described anecdotally. Overall, they have a wide range of clinical presentations and often overlap with each other leading to a delayed diagnosis. Achieving adequate control of skin toxicity in these cases often requires immunosuppressive systemic therapies and/or interruption of ICI treatment, presenting a therapeutic challenge in the context of cancer management. In this study, we present a case series from Italy based on a multicenter, retrospective, observational study, which included 45 patients treated with ICIs who developed ICI-induced bullous pemphigoid. In addition, we performed a comprehensive review to identify the cases reported in the literature on ICI-induced autoimmune bullous diseases. Several theories seeking their underlying pathogenesis have been reported and this work aims to better understand what is known so far on this issue.

## 1. Introduction

Immune checkpoint inhibitors (ICIs) have represented an innovation in the treatment of several malignancies since the approval of ipilimumab in 2011 (1). These are monoclonal antibodies targeting the cytotoxic T-lymphocyte antigen-4 (CTLA-4) or programmed cell death-1/ligand 1 (PD-1/PD-L1) which are involved in the negative regulation of T-cell immune function. This binding causes the failure of the tumoral evasion mechanisms, and, consequently, an increased triggering of the immune system against cancer. However, this immune activation is non-specific and it can affect many different organ systems leading to the so-called immune-related adverse events (irAEs) in up to 70% of treated patients, such as pneumonitis, colitis, endocrinopathies, and myocarditis (2).

The skin is the most involved organ being affected in approximately 30% of patients treated with anti PD-(L)1 and 50% with anti CTLA-4 drug, respectively (3, 4). Furthermore, patients who develop a cutaneous irAE (cirAE) need close monitoring for signs or symptoms of extracutaneous ones as it may be a predictive factor (5). It has been proposed to divide the cirAEs into four histopathological categories which are inflammatory, immunobullous, keratinocyte changes and melanocyte changes. The inflammatory ones are the most frequent and appear as maculopapular, lichenoid, psoriatic and eczematous eruptions (6). Even though the development of cirAEs has been associated with an increased survival and tumor response (7–9) their prognostic significance remains unclear (10).

Immunobullous eruptions have been increasingly reported in the literature mostly linked to anti PD-1/PD-L1 drugs. The estimated overall incidence varies from 1 to 5% (11, 12), with bullous pemphigoid (BP) being the most commonly observed phenotype. Lichen planus pemphigoides (LPP), pemphigus vulgaris (PV), and mucous membrane pemphigoid (MMP) have also been described in association with ICIs, albeit uncommonly. Bullous irAEs represent a therapeutic challenge for clinicians because they might result in significant morbidity and mortality if untreated. Moreover, immunosuppressive systemic therapies and/or ICI interruption are often required to reach adequate control of the cutaneous involvement resulting in a worsening of the cancer prognosis (11).

Herein, we reported our Italian case series about ICI-induced autoimmune blistering disorders, especially ICI-BP. In addition, we conducted a comprehensive review of bullous cirAEs to compare our data with the cases already described and to better understand what is known so far on this issue.

## 2. Materials and methods

This is a case series based on a national multicenter, retrospective, observational cohort including all patients treated with ICIs and developed an immunobullous cirAE during treatment or up to 12 months after discontinuation. Data were collected from 14 Italian hospitals between September 2021 and February 2023, after the institutional review board approval obtained from the ethic committee of the Turin University hospital. Information reported included patient demographics (age, sex), oncology history, ICI therapy, cirAE presentation and severity, histopathological findings, direct and indirect immunofluorescence (DIF/IIF), antibodies detected by enzyme-linked immunosorbent assay (ELISA), cirAE treatment, and tumor outcome. No patient identifiable data were used. The immunobullous cirAE diagnosis had to be supported by at least one positive test including histopathological examination, DIF, IIF or ELISA. Common Terminology Criteria for Adverse Events (CTCAE) ver. 5.0 was used to identify skin toxicity severity. Quantitative values were expressed as the median value and range.

Moreover, we performed a comprehensive review of the English-language medical literature about immunobullous cirAE. We used the databases PubMed, Embase, Scopus and Web of Science. Search strategy identified articles with the terms “bullous pemphigoid,” “lichen planus pemphigoides,” “pemphigus,” and “mucous membrane pemphigoid” combined with “cancer immunotherapy,” “immune checkpoint inhibitors,” “nivolumab,” “pembrolizumab,” “cemiplimab,” “ipilimumab,” “avelumab,” “atezolizumab,” “durvalumab.” The search involved all fields including title, abstract, keywords, and full text. Articles without available full text or with limited and inconsistent data from individual patients were excluded. We considered papers published by January 2023.

## 3. Immunobullous cirAEs–comprehensive review

### 3.1. Immune checkpoint inhibitor-induced BP and MMP

BP is characterized by subepithelial blister formation and inflammation with abundant eosinophils. Autoantibodies targeting two structural proteins of the dermal-epidermal junction (DEJ), BP antigen 1 (BPAG1 or BP230 antigen) and BPAG2 (or termed BP180 antigen), are involved in the pathogenesis (13). Its prevalence is increasing due to several factors such as the growing exposure to novel drug classes that might be implicated in eliciting the disease as the dipeptidyl peptidase 4 (DPP4) inhibitors and ICIs (13, 14). The reported incidence of BP as a cirAE varies between 0.3% and 3.8% across different studies (15–17).

**Figure 1:**
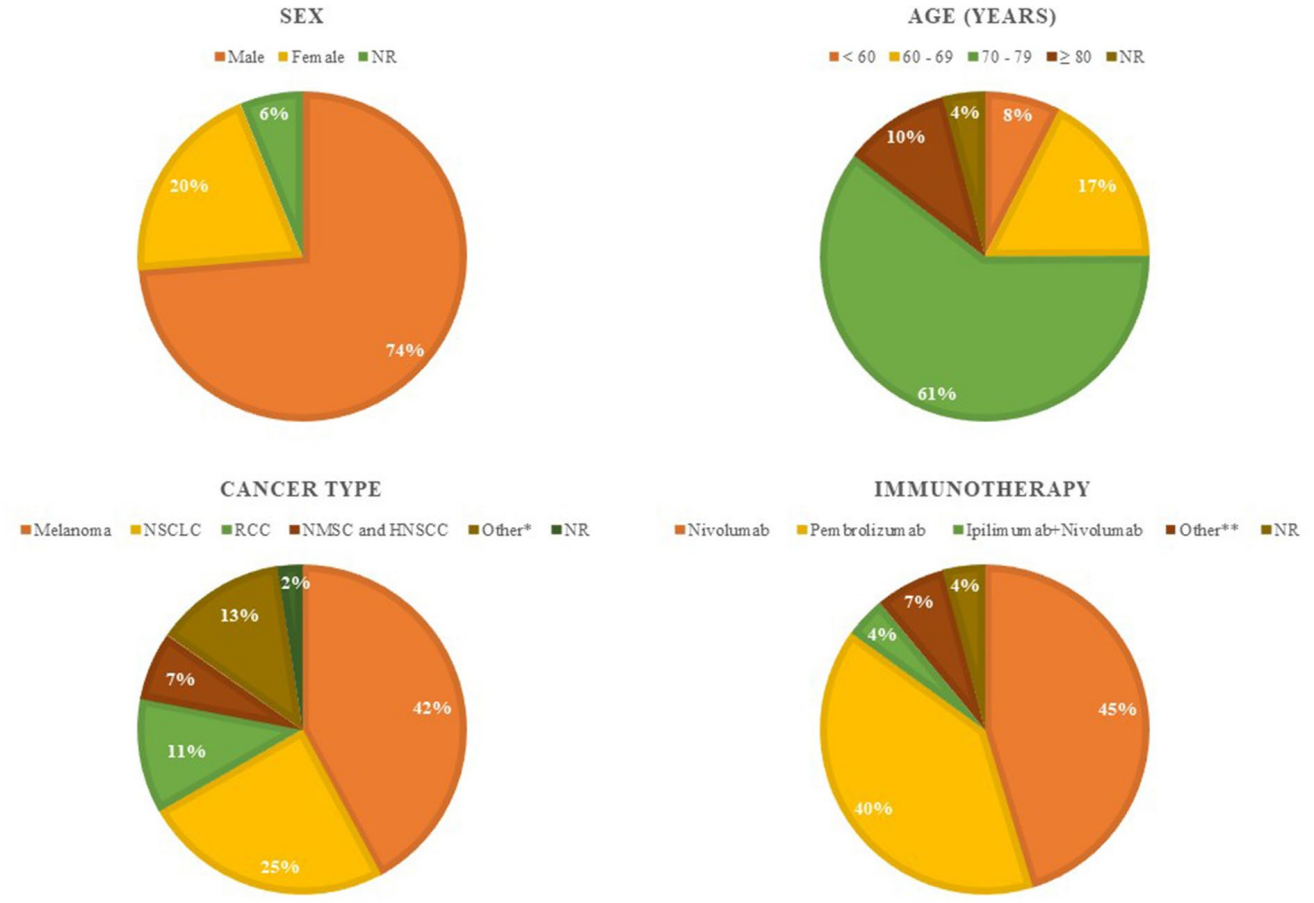
Demographic and clinical characteristics of the 373 ICI-induced BP cases identified among published articles. These pie charts report overall patient demographic [sex and age] and tumor characteristics [tumor type and immunotherapy]. *Other tumor type: urothelial cancer [*n* = 14], Merkel cell carcinoma [*n* = 4], colorectal cancer [*n* = 3], endometrial carcinoma [*n* = 2], breast cancer [*n* = 2], esophageal/gastric cancer [*n* = 2], mesothelioma [*n* = 2], prostate cancer [*n* = 2], thymoma [*n* = 1], hepatocellular carcinoma [*n* = 1], intrahepatic cholangiocarcinoma [*n* = 1], brain pinoblastoma [*n* = 1], glottic cancer [*n* = 1], salivary gland cancer [*n* = 1], peripheral T-cell lymphoma [*n* = 1], cervical cancer [*n* = 1], anaplastic thyroid carcinoma [*n* = 1]. **Other immunotherapy: cemiplimab [*n* = 7], durvalumab [*n* = 5], atezolizumab [*n* = 5], ipilimumab [*n* = 3], bintrafusp alfa [*n* = 4], tislelizumab [*n* = 1], avelumab [*n* = 1]. ICI, immune checkpoint inhibitor; BP, bullous pemphigoid; NR, not reported; NSCLC, non-small-cell lung cancer; RCC, renal cell carcinoma; NMSC, non-melanoma skin cancer; HNSCC, head and neck squamous cell carcinoma.

We collected the available data from 373 patients affected by ICI-induced BP and mainly published as case reports, case series and reviews (15, 16, 18–49). The collected information is summarized in Figure 1 and Table 1. The pie charts reported in Figure 1 identify the demographic and cancer characteristics. Men more frequently develop ICI-BP [275 of 373 (74%)] especially in the VII decade of life [226 of 373 (61%)]. The most common primary tumors were melanoma [157 of 373 (42%)] and NSCLC [92 of 373 (25%)], and anti PD-1 drug was the most frequently associated with this cirAE since nivolumab was implicated in 45% of cases (169 of 373) and pembrolizumab in 40% (148 of 373). Table 1 reports the details regarding ICI-BP features, as well as its management and the tumor outcome. The median time interval between ICI initiation and BP onset was 26 weeks (2–209). Although most BP cases developed during the administration of immunotherapy, 25 patients experienced BP onset after ICI discontinuation with a median interval of 9 weeks. According to the CTCAE ver. 5.0, among 202 patients with sufficient information, 43% (86 of 202) was affected by more than 30% of the body surface area and the mucosal involvement was reported in 20% of cases (76 of 373). Diagnosis of BP was established by biopsy and histopathological examination in 250 of 373 patients (67%). In several cases, additional tests confirmed the diagnosis including DIF (220 of 249 patients tested), IIF (107 of 144 patients tested), as well as ELISA and/or immunoblotting for BP180/BP230 autoantibodies. The levels of BP180 autoantibodies were elevated in 121 of the 172 patients tested (70%), while BP230 autoantibody levels were increased in only 27 of the 136 performed cases (20%). Immunotherapy was permanently discontinued after BP development in 49% of patients (182 of 373), and the most used treatment was systemic corticosteroids [231 of 373 (62%)] followed by tetracycline-class antibiotic associated or not with niacinamide/nicotinamide [140 of 373 (38%)]. Regarding the tumor outcome, among patients with available information (*n* = 218), 32% (*n* = 68) had stable disease, 23% (*n* = 51) had a complete response, 23% (*n* = 51) had progression disease, and 22% (*n* = 48) had a partial response.

**Table 1:** Clinical presentation, diagnosis, and management of the 373 ICI-induced BP cases identified among published articles.

Features	All patients (*N* = 373)	Features	All patients (*N* = 373)
Time interval between ICI initiation and BP onset, median in weeks (min - max)^A^	26 (2–209)	*Adjustment of ICI regimen*	*No.*
*Mucosal membrane involvement*	*No.*	Discontinued before BP onset, No., median interval in weeks between ICI interruption and BP onset	25, 9
Yes	76	Discontinued after BP onset	182
No	226	Temporarily discontinued then resumed	21
NR	71	Continued without interruption	76
*Highest CTCAE grade* ^B^	*No.*	NR	69
G1	33	*Treatments used for BP*	*No.*
G2	83	Topical steroid alone or no treatment	93
G3	69	Systemic steroid	231
G4	17	Tetracycline-class antibiotic alone, in association with niacinamide/nicotinamide	93, 47
Unable to determine	161	Dapsone	18
*Histopathologic examination*	*No.*	Dupilumab	15
Yes	250	Omalizumab alone, in association with IVIG	14, 2
No	34	Methotrexate	16
NR	89	Rituximab alone, in associationa with IVIG, in association with plasma exchange	14, 3, 1
*DIF*	*No.*	IVIG	9
Positive	220	Mycophenolate mofetil	8
Negative	29	Azathioprine	3
Not performed	23	Infliximab	1
NR	101	Acitretin	1
*IIF*	*No.*	Hydroxychloroquine	1
Positive	107	Ciclophosphamide	1
Negative	37	NR	23
Not performed	124	*Cancer outcome*	*No.*
NR	105	PD	51
*BP180 autoantibody*	*No.*	SD	68
Positive	121	PR	48
Negative	51	CR	51
Not measured	35	NR	155
NR	166		
*BP230 autoantibody*	*No.*		
Positive	27		
Negative	109		
Not measured	46		
NR	191		

This table summarizes the information about the latency of BP development and its CTCAE grade, diagnostic findings (histopathology, DIF, IIF, BP180 and BP230 autoantibodies), and management. ^A^Median time interval between ICI initiation and BP onset was calculated considering the weeks published in the article, or an estimation when the authors reported the number of treatment cycles/months. ^B^Highest ICI-BP grade is according to CTCAE ver. 5 (bullous dermatosis) and, if not reported by authors, was estimated from available clinical information; in 10 patients the grade reported was based on BPDAI score (mild in *n* = 2, moderate in *n* = 2, and severe in *n* = 6). ICI, immune checkpoint inhibitor; BP, bullous pemphigoid; CTCAE, Common Terminology Criteria for Adverse Events; DIF, direct immunofluorescence; IIF, indirect immunofluorescence; BPDAI, Bullous Pemphigoid Disease Area Index; NR, not reported; IVIG, intravenous immunoglobulin; CR, complete response; PR, partial response; SD, stable disease; PD, progression disease.

MMP is a pemphigoid disease with predominant mucosal involvement and cicatricial healing of its lesions. It is characterized by the production of autoantibodies directed against the C-terminal domain of BP180 combined or not with reactivity against the BP180-NC16A epitope. Other target antigens, such as BP230, laminin 332 a6b4-integrin and type VII collagen, have been identified (50). The oral cavity and conjunctiva are the most involved sites, following by nasopharynx and genitalia. The involvement of larynx, esophagus, and trachea can give life-threatening complications due to cicatricial strictures (51). Skin lesions can be present but are often confined to the face and scalp (52).

Among published literature, we identified 10 patients (5 males and 5 females), with an average age of 69 years (47–84 years), affected by ICI-induced MMP (33, 50, 52–58). Their characteristics are reported in Supplementary Table S1. Pembrolizumab was the mainly culprit ICI [6 of 10 (60%)] with a median time to onset since first administration of 33 weeks (3–66 weeks). MMP manifestations are generally classified according to the severity of the disease into low risk, defined as oral mucosa involvement with or without skin lesions, and high-risk, when any other site is involved resulting more frequently in cicatricial sequelae (58). Among this small case series, 80% of patients could be classified as low risk (33, 50, 52, 54–57) according to reported clinical information, while 20% as high risk (53, 58) considering the upper respiratory mucosa involvement that in one case required the tracheostomy due to laryngeal stenosis (53).

### 3.2. Immune checkpoint inhibitor-induced LPP

LPP was previously considered to be a variant of lichen planus (the so-called bullous LP) or BP. In fact, it would seem to be a distinct autoimmune subepidermal blistering disease characterized by the presence of autoantibodies targeting BP180 and a relatively benign course (59). The first clinical manifestation in both LPP and bullous LP is pruritic violaceous polygonal papules and plaques. Blisters and erosions appear later and mainly on the extremities. In LPP, bullous lesions typically develop both on unaffected and affected skin, while in bullous LP they appear on a previous lichenoid lesion (60). This clinical distinction does not occur in all cases making it necessary for the diagnosis the detection of anti BP180 autoantibodies as they are present in LPP but not in bullous LP. Indeed, it has been hypothesized that lichenoid inflammation itself may promote the development of an autoimmune response against DEJ in LPP, exposing several antigens due to extensive apoptosis of the basal epidermis. On the other hand, blisters occur in bullous LP as the result of a massive vacuolar degeneration of the basal keratinocytes, resulting in large dermal–epidermal separations (59). An association between the development of LPP and drugs or pre-existing medical conditions has been previously reported. In recent years, rare cases of anti-PD-1/PD-L1-induced LPP have been documented and we identified a total of 23 cases (11, 33, 61–77).

These patients are 13 females (57%) and 10 males (43%), with a median age of 66 years (12–87 years), older than that found in the classic type (median age of 46 years) (59). Complete data are reported in Supplementary Table S2. They received immunotherapy to treat especially lung cancer [9 out of 23 (39%)], melanoma [5 out of 23 (22%)] and renal cell/urothelial cancer [4 out of 23 (17%)]. Administered drugs included mainly pembrolizumab [12 out of 23 (52%)] and nivolumab [8 out of 23 (35%)]. In all reported cases, LPP began as a lichenoid dermatitis with or without blisters and with an average onset time of about 17 weeks (1 week–2 years) since the ICI initiation. In about 61% of patients (14 out of 23), the eruption was widespread affecting the trunk, upper and lower limbs. Mucosal involvement was reported in less than half of the patients [9 out of 23 (39%)], in most cases as erosive mucositis. Five cases did not manifest BP features to clinical (bullous lesions) and histological (subepidermal blisters containing eosinophils, perivascular mixed infiltrate) evaluation, therefore the diagnosis was made thanks to ELISA or immunoblotting. These tests revealed the presence of autoantibodies targeting BP180 in all the 16 patients tested. Treatment with immunotherapy was interrupted after LPP development in 16 patients (70%) and temporarily discontinued in 4 patients (17%). Local, oral and/or intravenous corticosteroids with a wide range of doses were the first-line therapy in all cases while 8 patients (35%) required other systemic therapies. Among patients with available data about tumor outcome (*n* = 15), 10 patients had progression disease at the last follow-up visit or died due to cancer, 4 patients had stable disease, while only one patient had a complete response.

### 3.3. Immune checkpoint inhibitor-induced PV and paraneoplastic pemphigus

Pemphigus is a group of life-threatening and rare blistering diseases characterized by the production of autoantibodies directed against intercellular adhesion molecules. These autoantibodies induce epidermal acantholysis leading to the formation of intraepidermal blisters that clinically manifest as flaccid bullae, erosions, pustules on the skin and/or mucosal erosions (78).

PV is the most common form, and it is associated with the production of autoantibodies directed against desmoglein 1 (Dsg1) and 3 (Dg3) (78). In several cases, it may be induced by drugs belonging to thiol, phenol, and non-thiol non-phenol classes, which may contribute to the development of acantholysis through several mechanisms (79). A few cases of pemphigus developed *de novo* or found to be aggravated upon introduction of immunotherapy have also been reported (78, 80–83) and we summarized their characteristics in Supplementary Tables S3, S4.

The immune mechanism that leads to the breakdown of tolerance in PV during therapy with ICIs are not fully understood. Schoenberg et al. described the case of a patient affected by ICI-PV and in whom investigation of the human leukocyte antigen (HLA) typing revealed three class II HLA alleles (DQB1*0302, DQA1*0301, and DRB1*04) associated with genetic susceptibility for pemphigus (81). This suggests that ICIs could unmask a genetic susceptibility stimulating the immune system and leading to the PV clinical expression. Furthermore, PV could be triggered in cancer patients by concomitant factors such as immunotherapy and radiotherapy (80, 84). Two cases of pre-existing PV recurred during immunotherapy have also been reported (82, 83). Patients with pre-existing autoimmune diseases have been excluded from the ICIs clinical trials due to the flare risk. Nevertheless, immunotherapy should not necessarily be ruled out in these patients as most relapses have been reported to be mild (85). Krammer et al. described the case of a PV-flare occurred during nivolumab therapy after a remission period of several years and resolved within 8 weeks of treatment with prednisolone tapering and methotrexate without requiring immunotherapy discontinuation (82). Therefore, a case of pre-existing pemphigus foliaceus not relapsed during ICI therapy has also been reported (86).

Paraneoplastic pemphigus (PNP) is another type of pemphigus whose clinical hallmark is recalcitrant and painful mucositis, which may be accompanied by polymorphic cutaneous eruptions as blisters, erosions, and lichenoid lesions. It is characterized by the production of autoantibodies against various target antigens, mainly envoplakin and periplakin (87), and occurs in the setting of occult or confirmed neoplasms, mostly lymphoproliferative disorders (up to 84% of reported cases) (88). There are also few cases of PNP occurred during immunotherapy (89–91) whose features suggest a relation between its onset and the oncological therapy instead of the cancer (Supplementary Tables S3, S4). In fact, it has been described in the setting of epithelial origin-carcinomas treated with ICIs, while they account for less than 10% of the classic PNP cases (88). Furthermore, McNally et al. reported a patient treated with pembrolizumab due to a urothelial carcinoma who developed PNP without evidence of active tumor. PNP is almost always associated with an active neoplasm, and it has rarely been reported in patients who are either in remission or have no detectable underlying neoplasm (90). Also the close temporal relation between ICI initiation and PNP onset suggests a triggering role of immunotherapy as reported in a patient who developed it after 3 months of pembrolizumab therapy due to a 10-year history of a SCC of the tongue (89).

## 4. Results–Italian case series

Table 2 summarizes the characteristics of the patients reported in our case series. These are 45 cases, 41 males (91%) and 4 females (9%), with a median age of 74 years (range 46–90). In all cases, they developed an ICI-induced BP while they were receiving the cancer treatment. The ICI identified were mainly nivolumab [28 of 45 (62%)] and pembrolizumab [11 of 45 (25%)], in the remaining cases combination ipilimumab with nivolumab, cemiplimab, spartalizumab, and atezolizumab were reported [6 of 45 (13%)]. They were used for the treatment of non-small-cell lung cancer (NSCLC) [18 of 45 (40%)], melanoma [12 of 45 (27%)], colorectal adenocarcinoma [5 of 45 (11%)], renal clear cell carcinoma [5 of 45 (11%)], head and neck squamous cell carcinoma (HNSCC) [4 of 45 (9%)], urothelial carcinoma [1 of 45 (2%)], and in 73% of cases they were stage IV (33 of 45). The median time to onset of cutaneous symptoms after ICI initiation was 35 weeks (range 4–260), while the median time to BP diagnosis was 48 weeks (range 5–286). In 19 patients (42%) pruritus without any cutaneous eruption was the first clinical manifestation, while bullous lesions appeared since the beginning in 9 patients (20%). The mucosal involvement was reported in 8 patients (18%). According to CTCAE grading for bullous dermatitis, BP affected more than 30% of the body surface in 40% of patients (G3 and G4 in 18 of 45 cases). Skin biopsy for histopathological examination was performed in 28 patients (62%) and it confirmed the diagnosis in all cases, while DIF and IIF were carried out in 31 (69%) and 26 (58%) patients, respectively, showing positive results in all cases. The ELISA for BP180 autoantibody was performed in 42 cases and was positive in 30 patients (66%), while the ELISA for BP230 autoantibody was carried out in 41 cases and was positive in 15 patients (33%). The first-line therapy of the cutaneous toxicity was topical steroids in 4 patients (9%), topical and systemic steroids in 41 (91%). In 18% of cases (*n* = 8) a second-line therapy was needed such as doxycycline (*n* = 5) and dapsone (*n* = 3). One patient required a third-line therapy with dupilumab. Immunotherapy was permanently discontinued for 17 patients (38%), while it was temporarily held for 16 patients (36%) of which about 50% (*n* = 7) experienced a relapse after rechallenging with the same ICI. The median time between the BP diagnosis and the control of symptoms was 9 weeks (range 1–68) in the 38 patients (84%) with a partial or complete response. In the remaining 16% (*n* = 7) the ICI-BP was refractory to the treatment. Tumor response of the 36 cases with available data revealed that 9 patients (20%) had a complete or partial response, 16 (36%) had stable disease, and 11 (24%) had progression disease.

**Table 2:** Characteristics of the 45 patients collected in our national multicenter cohort.

Characteristics	All patients (*N* = 45)	Characteristics	All patients (*N* = 45)
*Demographics*		*ICI-BP diagnosis*	
Sex, No. (%) male/female	41 (91)/4 (9)	*Histopathologic examination*	*No. (%)*
Age (years), median (range)	74 (46–90)	Yes	28 (62)
*Tumor type*	*No. (%)*	No	17 (38)
NSCLC	18 (40)	*DIF*	*No. (%)*
Melanoma	12 (27)	Yes	31 (69)
Colorectal adenocarcinoma	5 (11)	No	14 (31)
Renal clear cell carcinoma	5 (11)	*IIF*	*No. (%)*
HNSCC	4 (9)	Yes	26 (58)
Urothelial carcinoma	1 (2)	No	19 (42)
*Tumor stage*	*No. (%)*	*BP180 autoantibodies*	*No. (%)*
Stage IV	33 (73)	Positive	30 (66)
Stage III	9 (20)	Negative	12 (27)
Other or NR	3 (7)	Not performed	3 (7)
*Immunotherapy*	*No. (%)*	*BP230 autoantibodies*	*No. (%)*
Nivolumab	28 (62)	Positive	15 (33)
Pembrolizumab	11 (24)	Negative	26 (58)
Nivolumab + ipilimumab	2 (5)	Not performed	4 (9)
Cemiplimab	2 (5)	*ICI management*	*No. (%)*
Spartalizumab	1 (2)	ICI temporarily held	16 (36)
Atezolizumab	1 (2)	BP flare after rechallenged with the same ICI	7 (16)
*ICI-BP features*	*Median (range)*	ICI permanently discontinued	17 (38)
Time to symptoms onset after ICI initiation (weeks)	35 (4–260)	ICI-BP management	
Time to BP diagnosis after ICI initiation (weeks)	48 (5–286)	*First line therapy*	*No. (%)*
*First manifestations*	*No. (%)*	Topical costicosteroid	4 (9)
Pruritus without other manifestations	19 (42)	Topical corticosteroid + systemic corticosteroid	41 (91)
Eczematous eruption	11 (24)	*Second line therapy*	*No. (%)*
Bullous lesions	9 (20)	Doxycycline	5 (11)
Urticarial eruption	7 (16)	Dapsone	3 (7)
Mucositis	3 (7)	*Third line therapy*	*No. (%)*
Papular lesions	1 (2)	Dupilumab	1 (2)
*Mucosal membrane involvement*	*No. (%)*	*ICI-BP response*	*No. (%)*
No	37 (82)	Partial to complete resolution	38 (84)
Yes	8 (18)	Refractory symptoms	7 (16)
*CTCAE grade*	*No. (%)*	*Tumor response*	*No. (%)*
1	12 (27)	CR or PR	9 (20)
2	15 (33)	SD	16 (36)
3	17 (38)	PD	11 (24)
4	1 (2)	NR	9 (20)

All patients developed ICI-induced BP. The information reported is about patients’ demographics, primary cancer, immunotherapy, and ICI-induced BP. ICI, immune checkpoint inhibitor; BP, bullous pemphigoid; NSCLC, non-small-cell lung cancer; HNSCC, head and neck squamous cell carcinoma; NR, not reported; CTCAE, Common Terminology Criteria for Adverse Events; DIF, direct immunofluorescence; IIF, indirect immunofluorescence; CR, complete response; PR, partial response; SD, stable disease; PD, progression disease.

## 5. Discussion

Bullous autoimmune dermatoses are an uncommon cirAE whose prevalence is challenging to determine because it varies among several studies. Nevertheless, it is well known to be more infrequent than inflammatory eruptions or vitiligo (6, 92) since its reported incidence rates are less than 5% (11, 93). The exact underlying pathogenesis of bullous cirAEs has not fully understood but it potentially involves both the innate and adaptive immune systems.

In the tumor microenvironment, anti PD-1/PD-L1 antibodies enhance exhausted T effector cells function leading to immune activation against cancer. The lysis of tumoral cells releases numerous antigens whose presentation by antigen presenting cells causes the abnormal priming of both cytotoxic T lymphocytes and T helper 1 (Th1) cells. These mechanisms interfere with the immune tolerance resulting in the attack even against self-tissues (6). It is not surprising that lichenoid skin reaction is one of the most common cirAEs because this Th1-polarized reaction can cause intense interface dermatitis. It has been proposed that the lichenoid inflammation might expose antigens in the basal layer, making them targets for antibody development. In fact, research has demonstrated a role for T-cell trigger in enhancing the humoral response as well as a T-cell independent PD-1+ B-cell activation resulting in an aberrant antibodies production (94, 95). This theory could also explain why BP is the most common ICI-induced bullous dermatosis. In fact, the hemidesmosomes (BP180 and BP230) at the DEJ are more exposed than the desmogleins or other intercellular adhesion molecules to antibodies formation following the interface damage. In addition, the substantial male predominance among patients affected by ICI-induced BP, unlike classic BP, it could be associated with some sex-associated molecular differences. Indeed, it has been reported a higher tumor mutational burden and the presence of more immunogenic neoantigens in male patients with melanoma, so this may contribute to an increased incidence of irAE such as ICI-BP (96).

BP180 is normally expressed by undifferentiated keratinocytes of the basal layer, and it is ceased as they migrate upwards and differentiate in the epidermis. For this reason, it is expressed in SCC as a hallmark of de-differentiation (97). Therefore, it can also be detected in cells of neural crest origin as in proliferating melanocytes with an undifferentiated phenotype. Sequencing of COL17A1 from melanoma cDNA has revealed a series of aberrations that cause the post-translational degradation of the ectodomain and so its deficiency (98). Consequently, the endodomain accumulates in tumor cells, which has been associated with an invasive phenotype. This aberrant expression of BP180 is an accessible target for *in vitro* immunotherapy (99). These findings suggest that targeting of BP180 on tumor cells by the activated immune system could lead to a cross-reactive immunogenicity against the DEJ and the development of BP (100). The “same-antigen theory” could also explain ICI-induced PNP in patients affected by SCC as it is a cancer arising from keratinocytes and expressing autoantigens routinely identified in PNP (91). Moreover, anti-BP180 auto-antibodies in the sera of melanoma patients have been reported to be significantly higher than in the sera of healthy, at both early and advanced stages of disease, and this correlates with a higher probability for these patients to develop BP during anti-PD1 therapy (101).

The immunotherapy could also unmask a genetic susceptibility to develop bullous autoimmune disorders activating the immune system and leading to the clinical expression. The HLA-DQB1*03:01 allele which has been associated to BP has been found in higher frequencies in melanoma patients (102).

Data collected in our case series have confirmed what has already been described in the literature on ICI-induced BP. Unlike classic-BP, it has a male predominance (103) and this has been assumed to be associated with gender effects on immunotherapy activity (96). Drug-induced pemphigoid, as well as ICI-BP, is characterized by a younger age of onset (104) compared to the classic type whose incidence increases significantly over 80 years (105). BP-like eruption has been reported most frequently in patients receiving anti PD-1/PD-L1 antibodies for melanoma and NSCLC. It is not a class effect of these drugs but their mechanism of action close to peripheral tissues may be related to an increased reactivity against cutaneous self-antigens (106). ICI-BP appears later than other cirAEs (17, 107) and it is often preceded by a longer prodromal phase than classic BP characterized by persisting pruritus and/or non-specific dermatitis. In fact, it has a significantly longer delay from symptom onset to diagnosis than the classic one despite having similar delays from symptom onset to dermatology referral (47). Even though ICI-BP seems to have some peculiar clinical features, this does not reflect significant differences regarding histopathologic and DIF findings (35). Given the moderate-to-severe clinical presentation (17) and delayed diagnosis, management of ICI-BP often necessitates discontinuation of immunotherapy and treatment with oral/intravenous corticosteroids to control the cutaneous toxicity. Although several studies have suggested an association between the development of ICI-BP and improved cancer outcomes (7, 46, 48), the heterogeneity of information collected in this paper does not allow for confirmation of this theory. Future studies should evaluate the best tumor response in patients with ICI-BP based on cancer types and treatment modalities.

## 6. Conclusion

Bullous autoimmune dermatoses have gained increasing interest among cirAEs induced by anti PD-1/PD-L1 autoantibodies. Among the published literature, ICI-induced BP is the most frequently described, while LPP, MMP and pemphigus are reported anecdotally. There are several theories which try to clarify their underlying pathogenesis without a complete success. It potentially involves both the innate and adaptive immune systems, the cross-reactivity immunogenicity, the genetic susceptibility, and other unknown factors. The clinical presentation of these bullous cirAEs can vary, posing a challenge for prompt recognition and appropriate treatment. Immunossuppressive therapy and/or discontinuation of immunotherapy are often necessary for management. Dermatological referral is necessary to establish as soon as possible an appropriate therapeutic algorithm to control the cutaneous toxicity avoiding a negative impact on cancer outcome.

## Data availability statement

The original contributions presented in the study are included in the article/Supplementary material, further inquiries can be directed to the corresponding author.

## Author contributions

MM, SR, and PQ contributed to conception and design of the study and organized the database. MM and MAc contributed to literature search. MM, MR, AMa, GZ, FM, EA, RM, EC, AP, GG, PSo, CS, MC, LP, DF, RB, PSe, PV, MT, MAr, CV, AMi, RS, ED, and BM contributed to collection of the case series based on the national multicentre cohort. MM wrote the manuscript. MR, AMa, GZ, EA, EC, SR, and PQ contributed to manuscript revision. All authors read and approved the submitted version.

## Conflict of interest

The authors declare that the research was conducted in the absence of any commercial or financial relationships that could be construed as a potential conflict of interest.

## Publisher’s note

All claims expressed in this article are solely those of the authors and do not necessarily represent those of their affiliated organizations, or those of the publisher, the editors and the reviewers. Any product that may be evaluated in this article, or claim that may be made by its manufacturer, is not guaranteed or endorsed by the publisher.

## Supplementary material

The Supplementary material for this article can be found at: https://www.frontiersin.org/articles/10.3389/fmed.2023.1208418/full#supplementary-material
