# Supplementary material for: Autoimmune bullous dermatoses in cancer patients treated by immunotherapy: a literature review and Italian multicentric experience
Source: Front Med (Lausanne). 2023 Jul 20;10:1208418. doi: 10.3389/fmed.2023.1208418 (PMC10400335; doi:10.3389/fmed.2023.1208418)
Supplement: Supplementary file 1 [file Table_1.DOCX]

Supplementary Material

**Autoimmune bullous dermatoses in cancer patients treated by immunotherapy: a literature review and Italian multicentric experience**

Martina Merli^1*^, Martina Accorinti^1^, Maurizio Romagnuolo^2,3^, Angelo Marzano^2,3^, Giovanni Di Zenzo^4^, Francesco Moro^4^, Emiliano Antiga^5^, Roberto Maglie^5^, Emanuele Cozzani^6,7^, Aurora Parodi^6,7^, Giulia Gasparini^6,7^, Pietro Sollena^8^, Clara De Simone^8,9^, Marzia Caproni^10^, Luigi Pisano^11^, Davide Fattore^12^, Riccardo Balestri^13^, Paolo Sena^14^, Pamela Vezzoli^14^, Miriam Teoli^15^, Marco Ardigò^15^, Camilla Vassallo^16^, Andrea Michelerio^17,18^, Rosanna Rita Satta^19^, Emi Dika^20,21^, Barbara Melotti^22^, Simone Ribero^1†^ and Pietro Quaglino^1†^

^1^Dermatology Clinic, Department of Medical Sciences, University of Turin, Turin, Italy

^2^Dermatology Unit, Department of Internal Medicine, Fondazione IRCCS Ca' Granda Ospedale Maggiore Policlinico, Milan, Italy

^3^Department of Pathophysiology and Transplantation, Università degli Studi di Milano, Milan, Italy

^4^Laboratory of Molecular and Cell Biology, Istituto Dermopatico dell'Immacolata (IDI)-IRCCS, Rome, Italy

^5^Section of Dermatology, Department of Health Sciences, University of Florence, Florence, Italy

^6^Section of Dermatology, Department of Health Sciences (DISSAL), University of Genoa, Genoa, Italy

^7^Dermatology Unit, IRCCS Ospedale Policlinico San Martino, Genoa, Italy

^8^Dermatology Unit, Department of Surgical and Medical Sciences, Fondazione Policlinico Universitario A. Gemelli IRCCS, Rome, Italy

^9^Dermatology Unit, University Department of Medicine and Translational Surgery, Università Cattolica Del Sacro Cuore, Rome, Italy

^10^Immunopathology and Rare Skin Diseases Unit, Section of Dermatology, Department of Health Sciences, Azienda Unità Sanitaria Locale Toscana Centro, University of Florence, Florence, Italy

^11^Section of Dermatology, Department of Health Sciences, Azienda Unità Sanitaria Locale Toscana Centro, University of Florence, Florence, Italy

^12^Section of Dermatology, Department of Clinical Medicine and Surgery, Università degli studi di Napoli Federico II, Naples, Italy

^13^Division of Dermatology, Outpatient Consultation for Rare Diseases, APSS, Trento, Italy

^14^Dermatology Unit ASST-Papa Giovanni XXIII, Bergamo, Italy

^15^Porphyria and Rare Diseases, San Gallicano Dermatological Institute IRCCS, Rome, Italy

^16^Dermatology Clinic, Fondazione IRCCS Policlinico San Matteo, Pavia, Italy

^17^Dermatology Unit, Ospedale Cardinal Massaia, Asti, Italy

^18^Department of Clinical-Surgical, Diagnostic and Pediatric Sciences, University of Pavia, Pavia, Italy

^19^Department of Medical, Surgical, and Experimental Sciences, University of Sassari, Sassari, Italy

^20^Melanoma Center, Dermatology, IRCCS Azienda Ospedaliero-Universitaria di Bologna, Bologna, Italy

^21^Unit of Dermatology, Department of Medical and Surgical Sciences, DIMEC, Alma Mater Studiorum, University of Bologna, Bologna, Italy

^22^Oncology Unit, IRCCS Azienda Ospedaliero-Universitaria di Bologna, Bologna, Italy

*^†^ These authors share senior authorship*

*** Correspondence:** Martina Merli, MD**,** e-mail: [merlimartina93@gmail.com](mailto:merlimartina93@gmail.com)

# Supplemental Table 1

**Supplemental Table 1a**. This table reports the characteristics of the 10 patients identified from published articles on ICI-induced MMP. *Abbreviations: ICI, immune checkpoint inhibitor; MPP, mucous membrane pemphigoid; M, male; F, female; MCC, Merkel cell carcinoma; SCC, squamous cell carcinoma.*

| **First author, year (reference No.)** | **Age (years)** | **Sex** | **Tumour diagnosis** | **ICI drug** | **Time from ICI initiation to MPP onset** | **Clinical features** | **MPP treatment** | **ICI discontinuation** | **Tumour response** |
| --- | --- | --- | --- | --- | --- | --- | --- | --- | --- |
| Haug V, 2018 (1) | 62 | M | MCC | Pembrolizumab | 13 weeks | Erosions and blisters of the oral mucous membranes, especially the tongue and buccal mucosa. | Doxycycline, and topical corticosteroids | Yes | Partial response |
| Zumelzu C, 2018 (2) | 83 | F | Melanoma | Pembrolizumab | 66 weeks (24 weeks after ICI discontinuation) | Gingivitis with one tense blister, a large pseudomembrane-covered erosion with a tweezers sign, an atrophy and pseudo lichenoid lesions. | Topical corticosteroids and doxycycline | Yes  (not because of MMP) | Complete response |
| Sibaud V, 2019 (3) | 70 | F | Melanoma | Nivolumab | 12 weeks | Erythematous patches on the gingiva that progressed to desquamation with the formation of a pseudomembrane, erosions and oral ulcers. | Topical corticosteroids | No | Stable disease |
| Bezinelli LM, 2019 (4) | 47 | F | Ovarian clear cell adenocarcinoma | Pembrolizumab | 3 weeks | Multiple ulcerative lesions with hyperaemic borders of the oral mucosa; mild desquamation of the conjunctiva; erosion and fibrosis of the upper respiratory tract mucosa. | Topical corticosteroids, oral prednisone, infliximab, mycophenolate mofetil, intravenous methylprednisolone, rituximab, intravenous immunoglobulin; tracheostomy due to laryngeal stenosis. | Yes | Progression disease; death due to sepsis |
| Durmus O, 2020 (5) | 54 | F | Hodgkin's lymphoma | Nivolumab | 1 year | Extensive erosions with bright erythema on the maxillary and mandibular gingiva. | Topical corticosteroid, oral prednisone | No | - |
| Fässler M, 2020 (6) | 77 | M | Melanoma | Pembrolizumab | 6 months | Lichenoid papules on the limbs, oral erosions of the cheek mucosa and gingiva. | Doxycycline, nicotinamide, and topical corticosteroids | Yes  (not because of MMP) | - |
| Fässler M, 2020 (6) | 81 | F | Melanoma | Pembrolizumab | 6 months | Erosions of cheek mucosa, soft palate and desquamative gingivitis, isolated erosions on the face and scalp. | Doxycycline, oral corticosteroid, and topical corticosteroids | Yes  (not because of MMP) | - |
| Duan S, 2021 (7) | 75 | M | Urothelial carcinoma | Toripalimab | 5 months | Erosions on the dorsum of the tongue, buccal mucosa, and gingiva, with a few light white streaks and patches around. | Topical corticosteroids | Yes | - |
| Kawsar A, 2022 (8) | 59 | M | SCC | Durvalumab and tremelimumab | 12 months | Extensive erosions in oral mucosa. | Topical and oral/intravenous corticosteroids | Yes | Death to disease progression |
| Villaseca AL, 2023 (9) | 84 | M | Urothelial cancer | Pembrolizumab | 13.5 months | Oedema and erosive lesions in the soft palate and uvula, the tip and lingual surface of the epiglottis, aryepiglottic folds, and posterior pharyngeal wall. | Oral prednisone, methotrexate | Yes | Complete response |

**Supplemental Table 1b**. This table reports the characteristics of the 10 patients identified from published articles on ICI-induced MMP. *Abbreviations: ICI, immune checkpoint inhibitor; MPP, mucous membrane pemphigoid; DIF, direct immunofluorescence; IgG, immunoglobulins G; IgM, immunoglobulins M; C3, complement component 3; BMZ, basement membrane zone; IIF, indirect immunofluorescence; IgA, immunoglobulins A; ELISA, serum enzyme-linked immunosorbent assay.*

| **First author, year (reference No.)** | **Histopathologic findings** | **DIF** | **IIF** | **ELISA/Immunoblotting** |
| --- | --- | --- | --- | --- |
| Haug V, 2018 (1) | - | Linear C3 deposition along the BMZ | IgG and IgA deposition on epithelial roof of the cleavage | ELISA: anti-BP180 NC16A IgG positive  Immunoblots: IgG binding to the soluble 120-kDa ectodomain of BP180 (LAD-1) and IgG and IgA against the C-terminal segment 4575 of BP180 |
| Zumelzu C, 2018 (2) | Subepithelial cleavage with the overlying intact epithelium, a moderate perivascular infiltration consisting of lymphocytes and histiocytes. | Linear IgG and C3 deposition along the BMZ | - | IgG anti-BP180 e anti-230 negative |
| Sibaud V, 2019 (3) | Subepidermal cleft with a mixed inflammatory infiltrate composed predominately of lymphocytes and plasmocytes. | Linear IgG, IgA and C3 deposition along the BMZ | - | IgG anti-BP180 NC16A positive |
| Bezinelli LM, 2019 (4) | Subepithelial cleavage with an inflammatory infiltrate composed by neutrophils, eosinophils, and plasma cells, fibrosis in the lamina propria. | Linear IgG and non-specific granular IgM deposition along the BMZ | IgG deposition on the epithelial roof of the cleavage | Negative serum anti-laminin 332 autoantibodies |
| Durmus O, 2020 (5) | Subepidermal detachment with mixed inflammatory infiltrate. | Linear IgG deposition along the BMZ | - | - |
| Fässler M, 2020 (6) | Lichenoid inflammation with apoptotic basal keratinocytes (skin biopsy), subepithelial cleft formation with eosinophils (mucosa biopsy). | Linear IgG deposition along the BMZ | - | IgG anti-BP180 positive |
| Fässler M, 2020 (6) | Consistent with MMP. | Linear IgG and C3 deposition along the BMZ | - | IgG anti-BP180 and anti-BP230 negative |
| Duan S, 2021 (7) | Subepithelial cleft formation with proliferation and dilation of capillaries and dense neutrophils in the lamina propria. | Linear IgG and C3 deposition along the BMZ | - | IgG anti-BP180 positive |
| Kawsar A, 2022 (8) | - | - | - | IgG anti-BP180 e anti-230 positive |
| Villaseca AL, 2023 (9) | Ulcerated granulation tissue with mixed inflammation. | - | - | IgG anti-BP180 NC16A and desmoglein 1 positive; IgG anti-BP230 and desmoglein 3 negative |

# Supplemental Table 2

**Supplemental Table 2a**. This table reports the characteristics of the 23 patients identified from published articles on ICI-induced LPP. *Abbreviations*: *ICI, immune checkpoint inhibitor; LPP, lichen planus pemphigoides; M, male; F, female; NSCLC, non-small-cell lung cancer; SCC, squamous-cell carcinoma; BSA: body surface area.*

| **First author, year (reference No.)** | **Age (years)** | **Sex** | **Tumour diagnosis** | **ICI drug** | **Time from ICI initiation to LPP onset** | **Clinical features** | **Site** | **Mucositis** | **LPP treatment** | **ICI discontinuation** | **Tumour response** |
| --- | --- | --- | --- | --- | --- | --- | --- | --- | --- | --- | --- |
| Schmidgen MI, 2017 (10) | 64 | M | Melanoma | Pembrolizumab | 27 weeks | Earlier, pruritic, and erythematous papules and plaques with central vesicles. Six months later, blisters both on the lichenoid plaques and on unaffected skin. | Trunk, upper and lower extremities. | Yes | Topical clobetasol, systemic prednisolone, rituximab, PUVA therapy, acitretin, dapsone. | Yes | Complete response at the 2-year follow-up. |
| Siegel J, 2018 (11) | 64 | M | Melanoma | Nivolumab | 20 months | Violaceous papules with overlying scale in addition to scattered tense vesicles. | Trunk, upper and lower extremities. | No | High-potency topical steroid. | Yes  (temporarily) | Progression disease. |
| Strickley JD, 2019 (12) | 87 | F | NSCLC | Nivolumab | 12 weeks | Crusted erosions, flaccid bulla. | Trunk, upper and lower extremities. | No | Oral prednisone. | Yes | Stable disease at the 1-year follow-up. |
| Sato Y, 2019 (13) | 57 | M | NSCLC | Nivolumab | 24 weeks | Multiple erythematous papules and vesicles. | Trunk, upper and lower extremities. | No | Oral prednisolone, doxycycline. | Yes  (not because of LPP) | - |
| Lindner AK, 2019 (14) | 74 | M | Renal clear cell carcinoma | Nivolumab | 6 months | Erythematous papules and widespread severe pruritus. | Trunk. | Yes | Local and systemic steroids, acitretin. | Yes | Stable disease for 14 months. |
| Okada H, 2020 (15) | 76 | M | Urothelial cancer | Pembrolizumab | 12 weeks | Earlier, itchy erythematous patches and plaques with whitish scales. Three months later, tense blisters on unaffected skin. | Lower extremities. | Yes | Oral prednisolone. | Yes | Progression disease at the time of LPP diagnosis. |
| Kwon CW, 2020 (16) | 65 | F | Merkel cell  carcinoma | Pembrolizumab | 1 week | Diffuse pruritus, numerous erythematous-to-violaceous, hypertrophic, lichenified papules and plaques. | Trunk, upper and lower extremities. | No | Oral prednisone. | Yes | Stable disease after 15 months. |
| Senoo H, 2020 (17) | 76 | F | NSCLC | Atezolizumab | 16 weeks | Shiny, violaceous, flat-topped polygonal papules and plaques with Wickham’s striae accompanied by several vesicles. | Back, upper, and lower extremities. | Yes | Topical steroid. | Yes | Progression disease. |
| Kerkemeyer KLS, 2020 (18) | 75 | F | Lung cancer | Tislelizumab | 6 weeks | Widespread violaceous plaques with Wickham striae, erosions from prior blistering within and adjacent to the eruption. | Trunk, upper and lower extremities. | Yes | Topical betamethasone, oral prednisolone. | No | - |
| Manko S, 2021 (19) | 62 | F | SCC | Durvalumab | 2 years | Multiple erythematous, crusted, and excoriated papules. Two weeks later, extensive bullous lesions. | Trunk, face, upper and lower extremities. | Yes | Clobetasol cream, Narrow-band UVB phototherapy, oral prednisone. | Yes  (temporarily) | Durvalumab was resumed 2 months following bullous eruption due to a clinical progression of nodal metastasis. |
| Yoshida S, 2021 (20) | 70 | F | Melanoma | Pembrolizumab | 5 months | Multiple papules and erythematous plaques with scales, blisters. | Upper and lower extremities. | No | Oral prednisolone. | Yes | - |
| Sugawara A, 2021 (21) | 72 | F | Lung cancer | Pembrolizumab | 3 months | Earlier, pruritic purple-red papules and plaques. Later, oedematous erythema and tense blisters. | Trunk, upper and lower extremities. | No | Topical steroid, oral prednisolone. | Yes | Death due to disease progression. |
| Boyle MM, 2022 (22) | 66 | F | Urothelial cancer | Nivolumab | 4 weeks | Pink-violaceous papules and plaques with central erosions and vesicles, and flaccid bullae. | Nail, upper and lower extremities. | Yes | Oral prednisone, topical clobetasol 0.05% ointment, rituximab. | Yes | Disease progression. |
| Boyle MM, 2022 (22) | 59 | M | Hepatocellular carcinoma | Nivolumab | 48 weeks | Hyperkeratotic papules and plaques with overlying serous crusts, and tense and flaccid bullae. | Lower extremities. | No | Topical clobetasol 0.05% ointment. | Yes | Death due to disease progression. |
| Boyle MM, 2022 (22) | 57 | F | NSCLC | Pembrolizumab | 6 weeks | Papules and plaques with central hyperkeratosis. | Trunk, upper and lower extremities. | Yes | Oral prednisone, topical clobetasol 0.05% ointment. | Yes | Death due to disease progression. |
| Ee S, 2022 (23) | 84 | M | NSCLC | Pembrolizumab | 7 weeks | Purpuric papules and blisters. | Trunk, upper and lower extremities. | No | Topical clobetasol ointment. | No | - |
| Mueller KA, 2022 (24) | 12 | M | Spitzoid melanoma | Nivolumab | 6 weeks | Earlier, lichenoid papules coalescing into plaques, skin pain, joint swelling; later, rapidly spreading vesiculobullous lesions. | About 40% BSA involved. | No | Intravenous methylprednisolone, topical steroid ointment, acitretin, oral methotrexate. | Yes | Stable disease after 16 months since LPP diagnosis. |
| Qian J, 2022 (25) | 78 | M | Angiosarcoma | Pembrolizumab | 14 months | Pink papules coalescing into plaques, multiple 1-2 cm well-circumscribed ulcers and few intact bullae. | Trunk, upper and lower extremities. | No | Topical steroid, acitretin, oral prednisone. | Yes  (temporarily) | Pembrolizumab was re-started because of angiosarcoma progression. |
| Shah RR, 2022 (26) | 58 | F | Renal cell carcinoma | Nivolumab | 4 months | Violaceous plaques with polygonal configuration, tense blisters, and erosions. | Upper extremities. | No | Topical clobetasol cream, systemic corticosteroids, oral pregabalin. | Yes | - |
| Wat M, 2022 (27) | 80 | F | NSCLC | Pembrolizumab | 10 months | Earlier, pink to violaceous flat-topped papules. Subsequently, erythema with tense and eroded blisters. | Trunk, upper and lower extremities. | No | Intravenous corticosteroids, oral prednisone. | Yes | - |
| Wat M, 2022 (27) | 77 | M | NSCLC | Pembrolizumab | 6 months | Scaly erythematous papules coalescing into large plaques. |  | Yes | Steroid taper. | Yes | - |
| Wat M, 2022 (27) | 63 | F | Breast cancer | Pembrolizumab | 4 months | Red macerated plaques and flaccid bullae, papular eruption. | Trunk, upper and lower extremities. | No | Topical steroid, doxycycline, nicotinamide. | Yes  (temporarily) | - |
| Kawsar A, 2022 (8) | 64 | F | Ocular melanoma | Pembrolizumab | 2 months | Pruritus and bullae. | Lower extremities. | No | Topical and oral/intravenous corticosteroids. | Yes | Death due to disease progression. |

**Supplemental Table 2b**. This table reports the characteristics of the 23 patients identified from published articles on ICI-induced LPP. *Abbreviations: ICI, immune checkpoint inhibitor; LPP, lichen planus pemphigoides; DEJ, dermal epidermal junction; DIF, direct immunofluorescence; IgG, immunoglobulins G; C3, complement component 3; BMZ, basement membrane zone; IIF, indirect immunofluorescence; ELISA, serum enzyme-linked immunosorbent assay.*

| **First author, year (reference No.)** | **Histopathologic findings** | **DIF** | **IIF** | **ELISA/Immunoblotting** |
| --- | --- | --- | --- | --- |
| Schmidgen MI, 2017 (10) | Orthokeratosis, hypergranulosis, Civatte bodies, band-like lymphocytic infiltrate obscuring the DEJ. Subepidermal blister with eosinophils, dermal lymphocytic infiltrate. | Linear C3 deposition along the BMZ | Linear IgG deposition on epidermal side on salt-split skin | IgG anti-BP180 positive |
| Siegel J, 2018 (11) | Lichenoid interface dermatitis with eosinophils. | Negative | - | IgG anti-BP180 positive |
| Strickley JD, 2019 (12) | Band-like lichenoid mixed infiltrate consisting of lymphocytes, histocytes, and eosinophils. Subepidermal blister with a mixed infiltrate of lymphocytes and eosinophils. | Negative | Weak linear IgG deposition along BMZ on monkey oesophagus | - |
| Sato Y, 2019 (13) | Vacuolization of the basal layer with apoptotic keratinocytes, band-like infiltration of lymphocytes and eosinophils at DEJ. | Linear IgG deposition along the BMZ | - | IgG anti-BP180 positive |
| Lindner AK, 2019 (14) | Features of lichen ruber planus. | - | - | IgG anti-BP180 positive |
| Okada H, 2020 (15) | Orthokeratosis, irregular acanthosis, band-like lymphocytic infiltrate with vacuolar alteration. Subepidermal blister with eosinophilic infiltrates in the upper dermis. | Linear IgG and C3 deposition along the BMZ | - | IgG anti-BP180 positive |
| Kwon CW, 2020 (16) | Lichenoid and vacuolar interface alteration with dyskeratotic keratinocytes and eosinophils. | Linear IgG and C3 deposition along the BMZ | Linear IgG deposition on epidermal side of salt-split skin | IgG anti-BP180 positive |
| Senoo H, 2020 (17) | Hypergranulosis, vacuolar degeneration at the DEJ and band-like lymphocytic infiltration in the upper dermis. Subepidermal blister with mixed infiltrate of eosinophils and lymphocytes. | Linear IgG and C3 deposition along the BMZ | Negative | IgG anti-BP180 positive |
| Kerkemeyer KLS, 2020 (18) | Parakeratosis, acanthosis, elongation of rete ridges with basal cell degeneration. Subepidermal blister containing eosinophils, perivascular mixed inflammatory infiltrate with eosinophils. | Linear IgG and C3 deposition along the BMZ | - | - |
| Manko S, 2021 (19) | Lichenoid interface change, epidermal necrosis. Mixed inflammatory infiltrate in the papillary dermis composed of neutrophils, eosinophils, and lymphocytes, subepidermal bullae. | Linear IgG and C3 deposition along the BMZ | - | - |
| Yoshida S, 2021 (20) | Hyperkeratosis, sawtooth-like acanthosis, hypergranulosis, cytoid bodies, and lichenoid interface dermatitis with lymphocytic infiltration of the DEJ. Subepidermal blister and dermal lymphocytic infiltration. | Linear IgG and C3 deposition along the BMZ | IgG deposition on epidermal side of the salt-split skin | Immunoblotting revealed an IgG antibody specific for the C-terminus of BP180 |
| Sugawara A, 2021 (21) | Orthokeratosis, acanthosis and wedge-shaped hypergranulosis, vacuolar alteration of the basal layer and dermal lymphocyte infiltration. Subepidermal blister with eosinophilic infiltration. | IgG and C3 deposition along the BMZ | - | IgG anti-BP180 positive |
| Boyle MM, 2022 (22) | Orthokeratosis with hypergranulosis, lichenoid interface dermatitis with dyskeratotic keratinocytes at the lower epidermis. Subepidermal bulla, scattered eosinophils within the dermis. | Deposition of IgG and C3 along the BMZ with cytoid bodies and shaggy deposition of fibrinogen | - | IgG anti-BP180 and anti-BP230 positive |
| Boyle MM, 2022 (22) | Orthokeratosis, acanthosis, vacuolar interface dermatitis with dyskeratotic keratinocytes at the basal epidermis. Superficial to deep perivascular and interstitial inflammation with lymphocytes and numerous eosinophils within the dermis. | Linear deposition of IgG and C3 along the BMZ with cytoid bodies in the papillary dermis | - | - |
| Boyle MM, 2022 (22) | Orthokeratosis, focal parakeratosis, neutrophils in the stratum corneum, hypergranulosis, lichenoid interface dermatitis with dyskeratotic keratinocytes at the basal epidermis. | Linear IgG and C3 deposition along the BMZ | Linear IgG deposition along BMZ on monkey oesophagus | IgG anti-BP180 positive, anti-BP230 negative |
| Ee S, 2022 (23) | Basal vacuolar alteration with a few apoptotic keratinocytes and subepidermal clefting, lichenoid infiltrate of lymphocytes, histiocytes and eosinophils, melanin incontinence and melanophages within the upper dermis. | Linear IgG and C3 deposition along the BMZ | - | - |
| Mueller KA, 2022 (24) | Subepidermal split with spotty basal necrotic keratinocytes, sparse lichenoid lymphocytic inflammation, and scattered eosinophils. | Strong linear C3 and fibrinogen deposition | - | IgG anti-BP180 positive |
| Qian J, 2022 (25) | Irregular acanthosis with patchy hypergranulosis and orthokeratosis, moderate lichenoid infiltrate with scattered eosinophils. | Heavy IgG staining at the DEJ | - | IgG anti-BP180 and anti-BP230 positive |
| Shah RR, 2022 (26) | Acanthosis, band-like inflammatory infiltrate composed predominantly of lymphocytes with scattered eosinophils, vacuolar degeneration of the basal layer of the epidermis and scattered dyskeratotic keratinocytes. Subepidermal blister with an underlying sparse dermal perivascular infiltrate containing scattered eosinophils. | Linear deposition of C3 along the BMZ | - | - |
| Wat M, 2022 (27) | Acanthosis with a saw-toothed rete ridges pattern, lichenoid infiltrate composed of lymphocytes, histiocytes, and eosinophils, pigment-laden macrophages in the upper dermis. Subepidermal blister formation. | Linear C3 and IgG deposition along the BMZ | - | IgG anti-BP180 positive |
| Wat M, 2022 (27) | Hypertrophic epidermis with band-like lymphocytic infiltrate in the upper dermis. | Linear C3 and IgG deposition along the BMZ | - | IgG anti-BP180 positive, anti-BP230 negative |
| Wat M, 2022 (27) | Lichenoid tissue reaction with a lymphocytic infiltrate of the DEJ, vacuolar alteration and dyskeratosis. A subepidermal blister with scattered eosinophils. | - | - | IgG anti-BP180 positive |
| Kawsar A, 2022 (8) | Diffuse band-like inflammation. | Linear C3 and IgG deposition along the BMZ | - | - |

# Supplemental Table 3

**Supplemental Table 3**. This table reports the characteristics of the 5 patients identified from published articles on ICI-induced PV and PNP. *Abbreviations: ICI, immune checkpoint inhibitor; PV, pemphigus vulgaris; PNP, paraneoplastic pemphigus*; *M, male; F, female;* *SCC, squamous cell carcinoma; cirAE, cutaneous immune-related adverse event; DIF, direct immunofluorescence; Ig, immunoglobulins; IgG, immunoglobulins G; IgA, immunoglobulins A; C3, complement component 3; BMZ, basement membrane zone; IIF, indirect immunofluorescence; ELISA, serum enzyme-linked immunosorbent assay; Dsg1, desmoglein 1; Dsg3, desmoglein 3; Dsc2, desmocollin 2; Dsc3, desmocollin 3.*

| **First Author, Year (Reference No.)** | **Age (Years)** | **Sex** | **Tumour diagnosis** | **ICI drug** | **Bullous cirAE** | **Time from ICI initiation to PV/PNP onset** | **Clinical features** | **Site** | **Histopathologic findings** | **DIF and/or IIF** | **ELISA** | **Treatment** |
| --- | --- | --- | --- | --- | --- | --- | --- | --- | --- | --- | --- | --- |
| Ito M, 2018 (28) | 68 | M | Ureteral carcinoma | Nivolumab | PV | About 7 months | Bullae, pustules, and erosions. Koebner phenomenon. | Trunk, upper and lower extremities. | Neutrophilic pustules in the epidermis, infiltration of neutrophils and eosinophils in the upper dermis. | DIF: intercellular epidermal deposition of IgG and C3  IIF: circulating IgG anti-cell surface antibodies at a titre of 1:16 | Ig anti Dsg3, Dsc2, Dsc3 | Topical steroid, oral prednisone. |
| Chen WS, 2018 (29) | 75 | M | SCC of the tongue | Pembrolizumab | PNP-like features | Approximately 82 days | Urticarial plaques and tense blisters with erosions. | Trunk and lower extremities. | Acantholysis and apoptotic keratinocytes, suprabasal blister, eosinophilic spongiosis, lymphocytes and eosinophils in the dermis. | DIF: linear deposition of IgG and IgA along  the BMZ, and intercellular epidermal deposits of IgG and C3 | Ig anti BP230 | Discontinuation of Pembrolizumab, local steroid, oral prednisone. |
| Buquicchio R, 2021 (30) | 95 | M | Multiple and advanced SCCs of the head | Cemiplimab | PV | About 21 weeks | Large blisters, excoriations, and large necrotic haemorrhagic eschar. | Trunk, upper and lower extremities, head. | - | - | Ig anti Dsg1 | Discontinuation of Cemiplimab, oral prednisone, topical steroid. |
| McNally MA, 2021 (31) | 57 | F | Urothelial carcinoma of the bladder | Pembrolizumab | PNP | 9 months | Painful ulcers with extensive granulation. | Tongue, inferior vermillion lip, labia majora. | Lichenoid interface with subepithelial clefting. | DIF: linear deposition of immunoglobulins and complement along the BMZ and weak intercellular stain | Ig anti Dsg3, BP180, BP230 | Discontinuation of Pembrolizumab, oral prednisone, intralesional steroid, intravenous Ig. |
| Schoenberg E, 2021 (32) | 56 | F | Melanoma | First ipilimumab and nivolumab, then ipilimumab alone | PV | After each infusion of ipilimumab alone | Erythema with flaccid vesicles and superficial erosions. | Trunk and upper extremities. | Intraepidermal blister and acantholysis. | DIF: intercellular epithelial deposition of IgG and C3 | Ig anti Dsg1 and Dsg3 | Discontinuation of ipilimumab, topical steroid. |

# Supplemental Table 4

**Supplemental Table 4**. This table reports the characteristics of the 3 patients identified from published articles on PV and PNP aggravated by immunotherapy. *Abbreviations: PV, pemphigus vulgaris; PNP, paraneoplastic pemphigus; M, male; F, female; SCC, squamous cell carcinoma; ICI, immune checkpoint inhibitor; DIF, direct immunofluorescence; IgG, immunoglobulins G; C3, complement component 3; BMZ, basement membrane zone; IIF, indirect immunofluorescence; ELISA, serum enzyme-linked immunosorbent assay; Dsg1, desmoglein 1; Dsg3, desmoglein 3; Dsc1, desmocollin 1.*

| **First author, year (reference No.)** | **Age (years)**  **Sex** | **Tumour diagnosis**  **ICI drug** | **Pre-existing bullous autoimmune dermatosis** | **Time from ICI initiation to PV/PNP relapse** | **Clinical features** | **Histopathologic findings** | **DIF and/or IIF** | **ELISA and/or immunoblotting** | **Treatment** |
| --- | --- | --- | --- | --- | --- | --- | --- | --- | --- |
| Krammer S, 2019 (33) | 85  M | Lung adenocarcinoma  Nivolumab | **PV** diagnosed 14 years earlier and in remission under 5 mg daily of oral prednisolone. | About 1 year | Multiple superficial skin erosions and several blisters, discrete erosions of the oral mucosa. Nikolsky’s sign I (direct) and II (indirect) positive. | Suprabasal clefting, which turned into a blister with lumen filled with fibrin, acantholytic cells, eosinophils, and neutrophils. | DIF: intercellular deposits of IgG in the epidermis  IIF: positive | ELISA: Ig anti Dsg1 and Dsg3 | Topical steroid, oral prednisolone, methotrexate. |
| Yatim A, 2019 (34) | 64  M | Locally advanced cutaneous SCC complicating hidradenitis suppurativa  Pembrolizumab | **PNP** appeared in a subclinical form before the initiation of ICI therapy. | 3 weeks | Widespread cutaneous flaccid and tense blisters, pustular lesions, erosions, erythematous plaques. Erythema and oedema of the face. Bilateral pseudomembranous conjunctivitis and eyelid erosions. Severe stomatitis. Erosions of the glans penis, nasal mucosa, arytenoids, and the aryepiglottic folds. | First biopsy: acantholysis and intraepidermal blisters.  Second biopsy: spongiosis, exocytosis of eosinophils, intraepidermal pustules, necrotic keratinocytes, superficial perivascular dermal infiltrate of eosinophils and lymphocytes. | DIF: intercellular deposits of IgG in the epidermis, associated with linear deposits of C3 along the BMZ  IIF: positive | ELISA: negative  Immunoblotting: 250 kDa band corresponding to Dsc1 | Discontinuation of pembrolizumab (PNP relapse upon reintroduction), oral prednisone.  Death due to sepsis. |
| Clawson RC, 2021 (35) | 47  M | Glioblastoma multiforme  Nivolumab | **PV** diagnosed 6 years earlier and in remission after two cycles of rituximab. | 45 days | Multiple erosions of posterior oropharynx and gingiva. | - | - | ELISA: Ig anti Dsg3 | Administration of rituximab.  Death due to tumour progression. |

# References

1. Haug V, Behle V, Benoit S, Kneitz H, Schilling B, Goebeler M, et al. Pembrolizumab-associated mucous membrane pemphigoid in a patient with Merkel cell carcinoma. Br J Dermatol. 2018 Oct;179(4):993–4.

2. Zumelzu C, Alexandre M, Le Roux C, Weber P, Guyot A, Levy A, et al. Mucous Membrane Pemphigoid, Bullous Pemphigoid, and Anti-programmed Death-1/ Programmed Death-Ligand 1: A Case Report of an Elderly Woman With Mucous Membrane Pemphigoid Developing After Pembrolizumab Therapy for Metastatic Melanoma and Review of the Literature. Front Med (Lausanne). 2018;5:268.

3. Sibaud V, Vigarios E, Siegfried A, Bost C, Meyer N, Pages-Laurent C. Nivolumab-related mucous membrane pemphigoid. Eur J Cancer. 2019 Nov;121:172–6.

4. Bezinelli LM, Eduardo FP, Migliorati CA, Ferreira MH, Taranto P, Sales DB, et al. A Severe, Refractory Case of Mucous Membrane Pemphigoid After Treatment With Pembrolizumab: Brief Communication. Journal of Immunotherapy. 2019 Dec;42(9):359.

5. Durmus Ö, Gulseren D, Akdogan N, Gokoz O. Mucous membrane pemphigoid in a patient treated with nivolumab for Hodgkin’s lymphoma. Dermatol Ther. 2020 Nov;33(6):e14109.

6. Fässler M, Rammlmair A, Feldmeyer L, Suter VGA, Gloor AD, Horn M, et al. Mucous membrane pemphigoid and lichenoid reactions after immune checkpoint inhibitors: common pathomechanisms. J Eur Acad Dermatol Venereol. 2020 Feb;34(2):e112–5.

7. Duan S, Zhang X, Wang F, Shi Y, Wang J, Zeng X. Coexistence of oral mucous membrane pemphigoid and lichenoid drug reaction: a case of toripalimab-triggered and pembrolizumab-aggravated oral adverse events. Oral Surg Oral Med Oral Pathol Oral Radiol. 2021 Sep;132(3):e86–91.

8. Kawsar A, Edwards C, Patel P, Heywood RM, Gupta A, Mann J, et al. Checkpoint inhibitor-associated bullous cutaneous immune-related adverse events: a multicentre observational study. Br J Dermatol. 2022 Dec;187(6):981–7.

9. Lagos-Villaseca A, Koshkin VS, Kinet MJ, Rosen CA. Laryngeal Mucous Membrane Pemphigoid as an Immune-related Adverse Effect of Pembrolizumab Treatment. J Voice. 2023 Jan 18;S0892-1997(22)00429-5.

10. Schmidgen MI, Butsch F, Schadmand-Fischer S, Steinbrink K, Grabbe S, Weidenthaler-Barth B, et al. Pembrolizumab-induced lichen planus pemphigoides in a patient with metastatic melanoma. J Dtsch Dermatol Ges. 2017 Jul;15(7):742–5.

11. Siegel J, Totonchy M, Damsky W, Berk-Krauss J, Castiglione F, Sznol M, et al. Bullous disorders associated with anti-PD-1 and anti-PD-L1 therapy: A retrospective analysis evaluating the clinical and histopathologic features, frequency, and impact on cancer therapy. J Am Acad Dermatol. 2018 Dec;79(6):1081–8.

12. Strickley JD, Vence LM, Burton SK, Callen JP. Nivolumab-induced lichen planus pemphigoides. Cutis. 2019 Apr;103(4):224–6.

13. Sato Y, Fujimura T, Mizuashi M, Aiba S. Lichen planus pemphigoides developing from patient with non-small-cell lung cancer treated with nivolumab. J Dermatol. 2019 Oct;46(10):e374–5.

14. Lindner AK, Schachtner G, Tulchiner G, Staudacher N, Steinkohl F, Nguyen VA, et al. Immune-related lichenoid mucocutaneous erosions during anti-PD-1 immunotherapy in metastatic renal cell carcinoma - A case report. Urol Case Rep. 2019 Mar;23:1–2.

15. Okada H, Kamiya K, Murata S, Sugihara T, Sato A, Maekawa T, et al. Case of lichen planus pemphigoides after pembrolizumab therapy for advanced urothelial carcinoma. J Dermatol. 2020 Sep;47(9):e321–2.

16. Kwon CW, Murthy RK, Kudchadkar R, Stoff BK. Pembrolizumab-induced lichen planus pemphigoides in a patient with metastatic Merkel cell carcinoma. JAAD Case Rep. 2020 Oct;6(10):1045–7.

17. Senoo H, Kawakami Y, Yokoyama E, Yamasaki O, Morizane S. Atezolizumab-induced lichen planus pemphigoides in a patient with metastatic non-small-cell lung cancer. J Dermatol. 2020 Apr;47(4):e121–2.

18. Kerkemeyer KLS, Lai FYX, Mar A. Lichen planus pemphigoides during therapy with tislelizumab and sitravatinib in a patient with metastatic lung cancer. Australas J Dermatol. 2020 May;61(2):180–2.

19. Manko S, Côté B, Provost N. A case of durvalumab-induced lichenoid eruption evolving to bullous eruption after phototherapy: A case report. SAGE Open Med Case Rep. 2021;9:2050313X21993279.

20. Yoshida S, Shiraishi K, Yatsuzuka K, Mori H, Koga H, Ishii N, et al. Lichen planus pemphigoides with antibodies against the BP180 C-terminal domain induced by pembrolizumab in a melanoma patient. J Dermatol. 2021 Sep;48(9):e449–51.

21. Sugawara A, Koga H, Abe T, Ishii N, Nakama T. Lichen planus-like lesion preceding bullous pemphigoid development after programmed cell death protein-1 inhibitor treatment. J Dermatol. 2021 Mar;48(3):401–4.

22. Boyle MM, Ashi S, Puiu T, Reimer D, Sokumbi O, Soltani K, et al. Lichen Planus Pemphigoides Associated With PD-1 and PD-L1 Inhibitors: A Case Series and Review of the Literature. The American Journal of Dermatopathology. 2022 May;44(5):360.

23. Ee S, Liang MW, Tee SI, Wang DY. Lichen planus pemphigoides after pembrolizumab immunotherapy in an older man. Ann Acad Med Singap. 2022 Dec;51(12):804–6.

24. Mueller KA, Cordisco MR, Scott GA, Plovanich ME. A case of severe nivolumab-induced lichen planus pemphigoides in a child with metastatic spitzoid melanoma. Pediatr Dermatol. 2022 Jul 26;

25. Qian J, Kubicki SL, Curry JL, Jahan-Tigh R, Benjamin R, Heberton M, et al. Pembrolizumab-induced rash in a patient with angiosarcoma. JAAD Case Rep. 2022 Nov;29:21–4.

26. Shah RR, Bhate C, Hernandez A, Ho CH. Lichen planus pemphigoides: A unique form of bullous and lichenoid eruptions secondary to nivolumab. Dermatol Ther. 2022 May;35(5):e15432.

27. Wat M, Mollanazar NK, Ellebrecht CT, Forrestel A, Elenitsas R, Chu EY. Lichen-planus-pemphigoides-like reaction to PD-1 checkpoint blockade. J Cutan Pathol. 2022 Nov;49(11):978–87.

28. Ito M, Hoashi T, Endo Y, Kimura G, Kondo Y, Ishii N, et al. Atypical pemphigus developed in a patient with urothelial carcinoma treated with nivolumab. J Dermatol. 2019 Mar;46(3):e90–2.

29. Chen WS, Tetzlaff MT, Diwan H, Jahan-Tigh R, Diab A, Nelson K, et al. Suprabasal acantholytic dermatologic toxicities associated checkpoint inhibitor therapy: A spectrum of immune reactions from paraneoplastic pemphigus-like to Grover-like lesions. J Cutan Pathol. 2018 Oct;45(10):764–73.

30. Buquicchio R, Mastrandrea V, Strippoli S, Quaresmini D, Guida M, Filotico R. Case Report: Autoimmune Pemphigus Vulgaris in a Patient Treated With Cemiplimab for Multiple Locally Advanced Cutaneous Squamous Cell Carcinoma. Front Oncol. 2021;11:691980.

31. McNally MA, Vangipuram R, Campbell MT, Nagarajan P, Patel AB, Curry JL, et al. Paraneoplastic pemphigus manifesting in a patient treated with pembrolizumab for urothelial carcinoma. JAAD Case Rep. 2021 Apr;10:82–4.

32. Schoenberg E, Colombe B, Cha J, Orloff M, Shalabi D, Ross NA, et al. Pemphigus associated with ipilimumab therapy. Int J Dermatol. 2021 Aug;60(8):e331–3.

33. Krammer S, Krammer C, Salzer S, Bağci IS, French LE, Hartmann D. Recurrence of Pemphigus Vulgaris Under Nivolumab Therapy. Front Med (Lausanne). 2019;6:262.

34. Yatim A, Bohelay G, Grootenboer-Mignot S, Prost-Squarcioni C, Alexandre M, Le Roux-Villet C, et al. Paraneoplastic Pemphigus Revealed by Anti-programmed Death-1 Pembrolizumab Therapy for Cutaneous Squamous Cell Carcinoma Complicating Hidradenitis Suppurativa. Front Med (Lausanne). 2019;6:249.

35. Clawson RC, Tabata MM, Chen ST. Pemphigus vulgaris flare in a patient treated with nivolumab. Dermatol Ther. 2021 Mar;34(2):e14871.

**
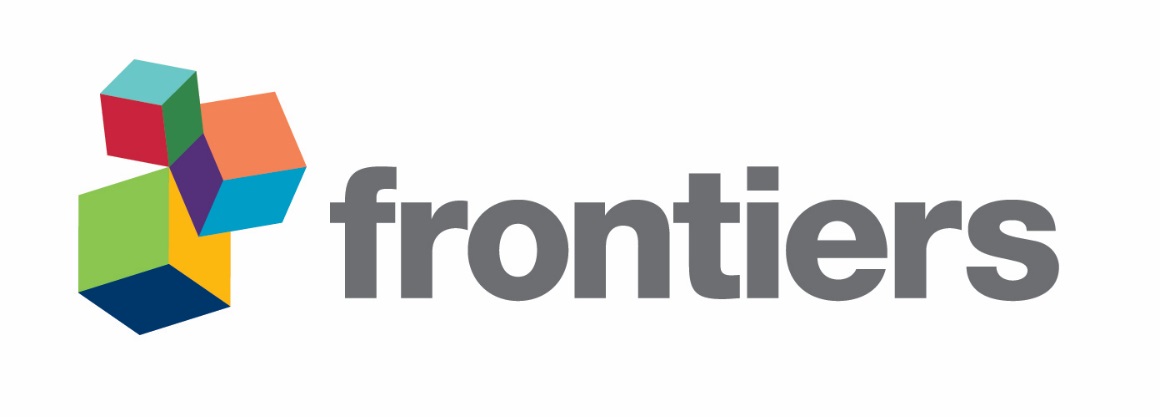
**
